# Activity levels and participation in physical activities by Korean patients following total knee arthroplasty

**DOI:** 10.1186/1471-2474-15-240

**Published:** 2014-07-17

**Authors:** Moon Jong Chang, Sung Hee Kim, Yeon Gwi Kang, Chong Bum Chang, Tae Kyun Kim

**Affiliations:** 1 Department of Orthopedic Surgery, Samsung Medical Center, Sungkyunkwan University School of Medicine, #81, Irwon-Ro, Gangnam-gu, Seoul 135-710, Republic of Korea; 2 Joint Reconstruction Center, Seoul National University Bundang Hospital, 166 Gumi-ro, Bundang-gu, Seongnam, Gyeonggi-do 463-707, Republic of Korea; 3 Department of Orthopaedic Surger, Seoul National University College of Medicine, Seoul National University Boramae Hospital, 5 Gil 20, Boramae-road, Dongjak-gu, Seoul 156-707, Republic of Korea; 4 Department of Orthopaedic Surgery, Seoul National University College of Medicine, Seoul, Republic of Korea

**Keywords:** Total knee arthroplasty, Physical activity, Patient factor, Satisfaction

## Abstract

**Background:**

The objectives of this study were to describe changes in physical activity profiles of Korean patients after TKA and to determine whether the postoperative physical activity level is influenced by patient socio-demographic factors and postoperative functional outcomes. We also sought to determine whether regular postoperative physical activity is associated with greater patient satisfaction after TKA.

**Methods:**

This observational study included 369 patients. Physical activity profiles before and after TKA were evaluated using a questionnaire that contained the UCLA activity scale and types of sports activities. The associations of socio-demographic features and postoperative functional outcomes with the physical activity levels were assessed using subgroup comparisons and partial correlation analyses. In addition, the effects of regular physical activity on patient satisfaction with replaced knees were evaluated using subgroup comparisons.

**Results:**

Walking, swimming and bicycling were the three most common sports activities both before and after TKA. After TKA, the mean activity level remained similar (UCLA score = 4.5 before TKA vs. 4.8 after TKA); however, the frequency of moderate activity levels (UCLA scale, 4-6) and moderate types of physical activities increased. Patients with higher postoperative function scores reported higher postoperative activity levels, but socio-demographic factors were not associated with activity level. Regular physical activity was associated with greater patient satisfaction.

**Conclusions:**

This study provides valuable information about realistic expectations for physical activity after TKA. Regular participation in physical activity should be encouraged to improve patient satisfaction.

## Background

Participation in physical activity can improve both general health status and postoperative satisfaction after total knee arthroplasty (TKA). Previously, surgeons usually recommended only sedentary activities after TKA because of concern about early implant failure [1]. However, due to the introduction of modern surgical techniques and prostheses, surgeons may now recommend more participation in physical activity. After TKA, the level of physical activity can be influenced by multiple factors including socio-demographic characteristics, postoperative pain and functional status. However, information about physical activity profiles before and after TKA and factors that influence physical activity is lacking. Furthermore, patients who regularly participate in physical activity after TKA may be more satisfied with the surgical outcome than less active patients. However, no studies have examined the relationship between physical activities and the level of postoperative satisfaction in non-selected patients after TKA.

Thus, this study was undertaken to describe the physical activity profiles of Korean patients before and after TKA and to determine whether socio-demographic factors and postoperative functional status influence postoperative activity levels. We also sought to determine whether regular physical activity is associated with patient postoperative satisfaction. We hypothesized that patients are more physically active postoperatively, that socio-demographic factors and postoperative functional status influence physical activity levels and that regular physical activity is associated with greater postoperative satisfaction.

## Methods

**Figure 1:**
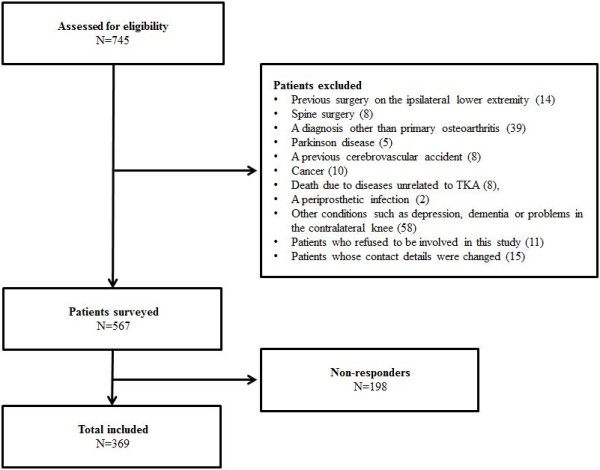
**Flowchart detailing inclusion and exclusion criteria of patients.** In total, 567 patients were included as candidates and were mailed the postal questionnaire. Of these patients, 369 (65%) returned the completed questionnaire or responded to a telephone survey (35 patients; 6%) at a mean of 2 years (range, 1 to 3 years) after surgery.

This study included 369 patients with TKA, and we performed a retrospective analysis of prospectively collected data. The inclusion criteria were: a diagnosis of osteoarthritis, TKA with follow-up care and the TKA was performed from 1 to 3 years before the survey. In total, 567 patients were included as candidates and were mailed the postal questionnaire. Of these patients, 369 (65%) returned the completed questionnaire or responded to a telephone survey (35 patients; 6%) at a mean of 2 years (range, 1 to 3 years) after surgery (Figure 1). There were 339 (92%) female patients and 30 (8%) male patients. The mean age was 68.8 years (range, 50–83 years). The mean preoperative height and weight were 153.3 cm (range; 140–179 cm) and 64.5 kg (range, 42–92 kg), respectively. The mean body mass index (BMI) was 27.4 kg/m^2^ (range, 19.3–39.1 kg/m^2^). The bilateral procedure was performed in 251 (68%) patients and the unilateral procedure in 118 (32%) patients.

The questionnaire consisted of these three parts (Additional file 1); Part I collected socio-demographic data, Part II evaluated the physical activities of patients before and after TKA, and Part III assessed patient satisfaction with overall surgical outcome and with their physical activity level. Part I included 4 questions that asked about place of residence (urban vs. rural), residence with family members, socioeconomic class (low, middle, high) and education level (no formal education, elementary school, middle school, high school, university or graduate school). Part II contained 5 questions about their regular physical activities before and after surgery, the types of physical activities in which the patient participated before and after surgery, and the preoperative and postoperative University of California at Los Angeles (UCLA) activity scale. They were asked about participation in the following 12 physical activities: hiking, swimming, cycling, walking, running, gymnastics, table tennis, badminton, gate ball, golf, stretching and other activities. The aforementioned activities were based on the 1999 Knee Society Survey of activity after TKA and adapted to the Korean cultural background [2]. Patients were also asked to list other activities in an open-ended question. We used the UCLA activity scale to assess patient activity level because it is a validated method for examining routine activity levels in a clinical series [3]. Furthermore, the UCLA activity scale provides the best reliability and has no floor effects [4]. Part III included 2 questions that evaluated patient satisfaction with the overall surgical outcome and with their physical activity level after surgery using a visual analog scale (VAS). The satisfaction scales ranged from 0 (indicating complete dissatisfaction) to 10 (indicating complete satisfaction). The reporting of the study follows the “Strengthening the Reporting of Observational studies in Epidemiology” (Additional file 2). All participants gave their informed consent to participate in the study, and this study was approved by the ethics committee of Seoul National University of Bundang Hospital.

An independent investigator (YGK) prospectively collected demographic characteristics and clinical data, which included height, weight, BMI, pain scores and function scores of the knee, using preprinted forms. Degrees of pain were measured using the pain subscale of the American Knee Society Score (AKS) [5] and the pain subscale of the Western Ontario and McMaster University Osteoarthritis Index (WOMAC) [6]. Functional status was measured using the function subscales of the AKS and WOMAC and SF-36 questionnaire [7]. Clinical evaluations were performed preoperatively and postoperatively at 6 months, 1 year and annually thereafter.

Statistical analysis was performed using SPSS for Windows (version 18.0; SPSS Inc., Chicago, IL), and P values of <0.05 were considered statistically significant. To describe the changes in the physical activity profiles following TKA, we compared the preoperative and postoperative UCLA activity score, the number of physical activities per person, the proportion of patients with regular physical activities and the types of physical activities, based on the questionnaire completed 1-3 years after TKA. The UCLA activity scores were summarized as means and ranges, and the statistical significance of the difference between the preoperative and the postoperative means was determined using the paired t test. We arbitrarily set the clinically meaningful difference at a difference greater than 1 point in the mean UCLA score because there were no previous studies suggesting a meaningful difference. In addition, the UCLA activity scores were categorized into low (≤3), moderate (4-6), and high (≥7) activity levels, and the preoperative and postoperative distributions these categories were compared using the McNemar’s test.

To determine whether patient socio-demographic factors and postoperative functional outcomes were associated with postoperative physical activity levels, subgroup comparisons and partial correlation analyses were performed. Socio-demographic data were dichotomized as follows: place of residence, urban vs. rural; living with family members, yes vs. no; education level, elementary school or lower vs. middle school or higher; and self-perceived income level, middle class or higher vs. low class. The mean UCLA score of the two groups, defined by the dichotomized socio-demographic factors, was compared using analysis of covariance (ANCOVA). The ANCOVA test was selected to adjust for the potential confounding effects of age, preoperative BMI and preoperative UCLA activity score, which were reported as factors that influence postoperative sports activities [8]. The effects of postoperative functional outcomes on physical activity levels were assessed using partial correlation analyses. The raw scores of the pain and functional scores of the WOMAC index, were transformed into a 0–100-point scale, with higher scores representing better outcomes. Age, preoperative BMI and UCLA activity scores were entered as covariates in the partial correlation analyses.

To determine whether regular participation in physical activity postoperatively was associated with patient satisfaction with replaced knees, the level of patient satisfaction of patients with and without regular physical activities was compared. The significance of differences in satisfaction VAS scores was determined using the ANCOVA test. To adjust for the potential confounding effects of potentially influential factors, the patient age, postoperative UCLA activity score, AKS function, WOMAC function, and the SF-36 PCS and MCS were entered as covariates in the ANCOVA test. We arbitrarily set the clinically meaningful difference at a difference greater than 1 point in VAS satisfaction score because no previous study has defined a clinically meaningful difference for the VAS satisfaction score.

## Results

Before and after TKA, walking, swimming and bicycling were the three most common physical activities both before and after TKA (Table 1), and the activity level after TKA remained similar (UCLA score = 4.5 before TKA vs. 4.8 after TKA) (Table 2). In addition, after TKA, the types and levels of physical activity appeared to converge toward the moderate. The mean UCLA activity score increased after surgery with statistical significance (p = 0.001), but the difference did not reach the level set for clinical significance (4.5 before TKA vs. 4.8 after TKA). The proportion of patients who participated in the three physical activity types regarded as moderate sports increased while that of patients participating in high impact sports, such as badminton and running, decreased (Table 1). Likewise, the proportion of patients with a moderate activity level increased after surgery (UCLA score 4–6) whereas patients with low activity (UCLA score ≤ 3) and highly active patients (UCLA scale ≥ 7) deceased after surgery (p < 0.001; Table 3). The mean number of physical activities per patient increased with statistical significance (p = 0.014), but the difference seemed to be of no clinical implication (1.5 before surgery and 1.7 after surgery).

**Table 1:** Types of sports activities before and after TKA

**Preoperative**	**Postoperative**
Walking 177 (48.0)	Walking 221 (59.9)
Swimming 79 (21.4)	Swimming 85 (23.0)
Cycling 60 (16.3)	Cycling 80 (21.7)
Hiking 34 (9.2)	Hiking 22 (6.0)
Stretching 17 (4.6)	Gymnastics 17 (4.6)
Gymnastics 14 (3.8)	Stretching 13 (3.5)
Badminton 9 (2.4)	Badminton 6 (1.6)
Running 7 (1.9)	Running 5 (1.4)
Golf 7 (1.9)	Gate ball 4 (1.1)
Table tennis 5 (1.4)	Table tennis 3 (0.8)
Gate ball 3 (0.8)	Golf 2 (0.5)
Others 10 (2.7)	Others 11 (3.0)

Data are presented as numbers (percentages).

**Table 2:** Preoperative and postoperative physical activity levels by UCLA scales

**Parameter**	**Preoperative**	**Postoperative**	***P *** **value**
Mean UCLA score	4.5 (Median, 4; SD, 1.8)	4.8 (Median, 4; SD, 1.4)	0.001
Category of UCLA score			< 0.001
UCLA score ≤ 3	90 (24%)	39 (11%)	
UCLA score 4–6	240 (65%)	296 (80%)	
UCLA score ≥ 7	39 (11%)	34 (9%)	

Abbreviation: UCLA scale, University of California at Los Angeles activity scale; SD, standard deviation.

**Table 3:** Comparisons of UCLA activity scores between groups categorized by socio-demographic factors

**Parameter**	**Postoperative UCLA score**			***P *** **value**
	**Mean**	**SD**	**Range**	
Place of residence				
Urban	4.9	1.4	2–10	0.376
Rural	4.7	1.5	2–10	
Living with family member				
Yes	4.9	1.4	2–10	0.918
No	4.6	1.4	2–10	
Education level				
≤ Elementary school	4.7	1.4	2–10	0.286
≥ Middle school	5.0	1.4	2–10	
Self perceived income level				
≥ Middle class	4.9	1.4	2–10	0.112
Low class	4.6	1.4	2–10	

Data were evaluated by analysis of covariance after adjustment for age, preoperative body mass index and preoperative UCLA score. Abbreviation: SD, standard deviation.

Postoperative activity levels were associated with postoperative function scores but not with socio-demographic factors, pain relief, and postoperative range of motion (Tables 3 and 4). Higher postoperative function scores, including WOMAC function (r = 0.149, p = 0.009), SF-36 PCS (r = 0.228, p < 0.001) and SF-36 MCS (r = 0.122, p = 0.032), were related to higher postoperative UCLA activity scores. Among these measures of function, the SF-36 PCS was most strongly correlated with the level of physical activity after surgery. Furthermore, AKS function scores also tended to be related to activity level after TKA (r = 0.108, p = 0.058). In contrast, no association was found between postoperative UCLA activity score and any patient socio-demographic factor, postoperative ROM or pain score (p > 0.05).

**Table 4:** Correlations between pain or function parameters and the postoperative UCLA activity score

**Parameter**	**Correlation coefficient**	***P *** **value**
Postoperative ROM	0.075	0.184
AKS		
Pain	0.056	0.326
Function	0.108	0.058
WOMAC		
Pain	0.064	0.260
Stiffness	0.043	0.441
Function	0.149	0.009
SF-36		
PCS	0.228	< 0.001
MCS	0.122	0.032

Data were evaluated by partial correlation analysis adjusted for age, preoperative BMI and preoperative UCLA scale. Abbreviations: AKS, American Knee Society Score; WOMAC, Western Ontario and McMaster University Osteoarthritis Index; PCS, physical component summary; MCS, mental component summary; ROM, range of motion.

Regular physical activity was associated with better patient satisfaction after TKA. The proportion of patients who participated in regular physical activities increased from 71% (260/369) before surgery to 76% (281/369) after surgery (p < 0.001). Patients who undertook regular physical activities after TKA reported higher satisfaction with the overall surgical outcome (7.9 vs. 7.2, p = 0.023) and with the physical activity level (7.5 vs. 6.3, p = 0.018) than the less active patients. However, the difference in overall satisfaction score did not reach the level of clinical significance, defined as a difference >1 point.

## Discussion

Participation in physical activity after TKA may improve general health status and influence patient satisfaction with replaced knees, but little is known about physical activities in patients with TKA. In this study, we described the physical activity profiles before and after TKA in Korean patients. We also determined whether patient socio-demographic factors and postoperative functional outcomes were associated with postoperative activity levels and whether regular physical activities were associated with patient satisfaction with replaced knees.

The present study has several limitations that warrant consideration. First, this study was performed using a questionnaire that was mailed 1 to 3 years after the patients underwent TKA. Because the patients had to recall their preoperative physical activities for the questionnaire, recall bias might exist. In an effort to reduce this bias, we included patients who had TKA within 3 years prior to the survey. In contrast, it was possible that some patients who underwent TKA 1 year before responding to the questionnaire had not achieved their final functional recovery yet. Second, 65% of patients responded to the questionnaire; thus, nonresponse bias might be present. However, we observed that non-responders did not differ from responders in demographic characteristics and postoperative health-related quality of life (SF-36) (data not presented), which suggests that nonresponse bias was minimal. Third, most study subjects were female (92%); thus, caution is required when comparing our findings to other cohorts with different sex compositions. For example, the mean postoperative UCLA score of the patients in this study was lower than that of previous studies [8,9]. This may be explained by the differences in gender compositions because a study reported that men had higher activity levels than women [10]. However, female predominance in TKA patients is common in Asians and Westerners and has been observed in Korean patients [11-14]. Therefore, despite the preponderance of female subjects in our study, our study may provide information valuable to clinicians, particularly those who care for patient populations with similar sex compositions.

Our findings refute the hypothesis that patients participate more actively in physical activities after TKA than they did before surgery. Our findings partly echo previous reports of declines in the actual rate of sports participation despite improvement in the UCLA activity score [8,9,15,16]. Furthermore, a previous study found that the mean number of sports per patient decreased from 1.8 to 1.4 after TKA [17]. In contrast, the number of physical activities per patient and the proportion of patients with regular physical activity increased in our study. These findings may be attributed to the fact that the majority of our patients participated in low-impact sports such as walking, cycling and swimming. A previous study found that after TKA patients were more likely to return to low-impact sports than to high-impact sports [16]. Another previous study reported that the largest decline after joint replacement occurred in high-impact sports, including badminton, tennis and dancing [18]. Our patients reported a similar pattern of decreased participation in high-impact sports, such as running and badminton. Our findings, taken together with previous studies, indicate that after TKA the activity levels of patients change to moderate levels because high impact physical activity decreases and low impact physical activities increases. These findings can contribute to prosthesis design, preoperative patient counseling and postoperative rehabilitation [8,9,15-18].

Initially, we hypothesized that postoperative activity levels would be affected by patient socio-demographic factors and postoperative functional status. Postoperative activity scores correlated with the functional domain scores of AKS and WOMAC and the physical and mental component summary scores of SF-36. These findings agree with reports of a positive association between postoperative functional scores and activity, although the levels of correlation we observed differed from those of previous studies [10,19]. Previously, the correlation coefficient between UCLA score and functional domains of AKS, WOMAC or PCS of SF-12 were reported as -0.50, 0.51 and 0.46, respectively [10]. These values are greater than ours. We cannot fully explain these discrepancies, but this may be partly caused by differences in ethnicity or cultural background. Our findings suggest that the activity levels after TKA may be influenced by postoperative functional status rather than by patient socio-demographic features.

This study confirms our hypothesis that patients who undertake regular physical activity have greater postoperative satisfaction. Patients with regular sports activities were more satisfied with both overall surgical outcomes and physical activity levels. Although differences in the types of sports and patient populations limit direct comparisons, our findings are in accordance with previous studies of the level of satisfaction in patients playing high-demand sports activities [1,20]. A study found that the mean satisfaction score was remarkably high (9.1 in 0-10 VAS scale) in patients who participated in high-demand sports after TKA [20]. Another study found that all patients who played tennis after TKA were satisfied with their surgical outcomes [1]. Although a pain-free knee joint with improved function is likely paramount to greater satisfaction after surgery, our results suggest that regular postoperative physical activity should be recommended to TKA patients to increase their satisfaction with the results of the surgery.

## Conclusions

This study described the physical activity profiles after TKA in Korean patients. Walking, swimming and bicycling were the three most common sports activities both before and after TKA. After TKA, the mean activity level remained similar; however, moderate activity levels and moderate types of physical activities increased. Activity levels are mainly influenced by postoperative function status and not by socio-demographic factors. These findings should be considered in prosthesis design, preoperative patient counseling and postoperative rehabilitation. Regular participation in physical activity should be encouraged to improve patient satisfaction with the results of the surgery.

## Abbreviations

AKS: American knee society; ANCOVA: Analysis of covariance; BMI: Body mass index; MCS: Mental component summary; PCS: Physical component summary; SF-36: Short form-36; TKA: Total knee arthroplasty; UCLA: University of California at Los Angeles; VAS: Visual analogue scale; WOMAC: Western Ontario and McMaster University Osteoarthritis Index.

## Competing interests

The authors declare that they have no competing interests.

## Authors’ contributions

MJC participated in the study design and helped write the manuscript. SHK helped with the postal survey. YGK performed the statistical analysis. CBC participated in the design of the study. TKK conceived of the study, and participated in its design. All authors read and approved the final manuscript.

## Pre-publication history

The pre-publication history for this paper can be accessed here:

http://www.biomedcentral.com/1471-2474/15/240/prepub

## Supplementary Material

Additional file 1 Questionnaire for postal survey. Click here for file

Additional file 2 STROBE Statement. Click here for file
