# Supplementary material for: Activity levels and participation in physical activities by Korean patients following total knee arthroplasty
Source: BMC Musculoskelet Disord. 2014 Jul 17;15:240. doi: 10.1186/1471-2474-15-240 (PMC4108970; doi:10.1186/1471-2474-15-240)
Supplement: Additional file 1 — Questionnaire for postal survey. [file 1471-2474-15-240-S1.pdf]

Appendix A

Part I of the questionnaire

1. Please check the location in which you live.

□ Urban area □ Rural area □ Other (specify :)

2. Please check your usual sitting style.

□ Korean (usually sit on the floor)

□ Western (usually sit on a chair)

3. Do you live with a family member?

□ Yes □ No

4. Please check your education level.

□ No formal education

□ Elementary school

□ Middle school

□ High school

□ University or graduate school

5. Please check your subjective income level.

□ Low □ Middle □ High

Appendix B

Part II of the questionnaire

Please answer the following questions about your sports activities before and after surgery.

1. Did you play sports regularly before surgery?

□ Yes □ No

2. If you played sports before surgery, please check the types of activities.

□ Hiking, □ Swimming, □ Cycling, □ Walking, □ Running, □ Gymnastics, □Table tennis, □ Badminton, □ Gate ball, □ Golf, □ Tennis, □ Stretching, □ Other (specify :)

3. Please check your level of activity before surgery. (UCLA activity scale)

□ Regularly participates in impact sports such as jogging, tennis, skiing, acrobatics, ballet, heavy labor, or back packing

□ Sometimes participates in impact sports

□ Regularly participates in very active events, such as bowling or golf

□ Regularly participates in active events, such as bicycling

□ Regularly participates in moderate activities, such as swimming and unlimited house work or shopping

□ Sometimes participates in moderate activity

□ Regularly participates in mild activities, such as walking, limited house work, and limited shopping

□ Sometimes participates in mild activity

□ Mostly inactive: restricted to minimal activities or daily living

□ Wholly inactive: dependent on others; cannot leave residence

4. If you played sports before surgery, were you able to resume your preoperative sports activities after surgery?

□ Yes □ No

5. Do you play sports regularly after the surgery?

□ Yes □ No

6. If you play sports activities after the surgery, please check the types of activities.

□ Hiking, □ Swimming, □ Cycling, □ Walking, □ Running, □ Gymnastics, □ Table tennis, □ Badminton, □ Gate ball, □ Golf, □ Tennis, □ Stretching, □ Other (specify :)

7. Please check your level of activity after surgery. (UCLA activity scale)

□ Regularly participates in impact sports such as jogging, tennis, skiing, acrobatics, ballet, heavy labor, or back packing

□ Sometimes participates in impact sports

□ Regularly participates in very active events, such as bowling or golf

□ Regularly participates in active events, such as bicycling

□ Regularly participates in moderate activities, such as swimming and unlimited house work or shopping

□ Sometimes participates in moderate activity

□ Regularly participates in mild activities, such as walking, limited house work, and limited shopping

□ Sometimes participates in mild activity

□ Mostly inactive: restricted to minimal activities or daily living

□ Wholly inactive: dependent on others; cannot leave residence

8. If you do not play sports activities after surgery, please check the causes.

□ Problems in the operated knee

□ Problems in the non-operated knee

□ Lack of sports facilities

□ Comorbidities not related to the knee joint

□ a. Other joint problems

□ b. Cardiovascular diseases (hypertension, myocardial infarction, etc)

□ c. Respiratory diseases (asthma, etc)

□ d. Endocrine diseases (diabetes, etc)

□ e. Other (specify:)

□ f. Other (specify:)

Appendix C

Part III of the questionnaire

Please answer the following questions about your level of satisfaction after surgery.

1. Please rate your level of satisfaction with the overall surgical outcome.

□ Very satisfied

□ Satisfied

□ Neither satisfied nor dissatisfied

□ Dissatisfied

□ Very dissatisfied

2. Please rate your level of satisfaction with the overall surgical outcome.

Very dissatisfied 0 1 2 3 4 5 6 7 8 9 10 very satisfied

3. How do you rate your current physical activity as compared with your preoperative expectation?

□ Better than expected □ As expected □ Poorer than expected

4. How do you rate your current physical activity as compared with peers?

□ Better □ Equal □ Poorer

5. Please rate your level of satisfaction regarding your level of physical activity.

Very dissatisfied 0 1 2 3 4 5 6 7 8 9 10 very satisfied
