# Supplementary material for: Activity levels and participation in physical activities by Korean patients following total knee arthroplasty
Source: BMC Musculoskelet Disord. 2014 Jul 17;15:240. doi: 10.1186/1471-2474-15-240 (PMC4108970; doi:10.1186/1471-2474-15-240)
Supplement: Additional file 2 — STROBE Statement. [file 1471-2474-15-240-S2.pdf]

STROBE Statement

	Item No	Recommendation
**Title and abstract**	1	(*a*) Indicate the study's design with a commonly used term in the title or the abstract<br>- Abstract; line 7
		(*b*) Provide in the abstract an informative and balanced summary of what was done and what was found<br>- Abstract; line 7-20
**Introduction**		
Background/rationale	2	Explain the scientific background and rationale for the investigation being reported<br>- Background; line 26-37
Objectives	3	State specific objectives, including any prespecified hypotheses<br>- Background; line 38-44
**Methods**		
Study design	4	Present key elements of study design early in the paper<br>- Methods; line 47-48
Setting	5	Describe the setting, locations, and relevant dates, including periods of recruitment, exposure, follow-up, and data collection<br>- Methods; line 48-52
Participants	6	(*a*) Give the eligibility criteria, and the sources and methods of selection of participants<br>- Methods; line 48-49
Variables	7	Clearly define all outcomes, exposures, predictors, potential confounders, and effect modifiers. Give diagnostic criteria, if applicable<br>- Methods; line 58-89
Data sources/ measurement	8*	For each variable of interest, give sources of data and details of methods of assessment (measurement). Describe comparability of assessment methods if there is more than one group<br>- Methods; line 58-89
Bias	9	Describe any efforts to address potential sources of bias<br>- Methods; line 49, Discussion; line 173-174
Study size	10	Explain how the study size was arrived at<br>- Methods; line 48-49
Quantitative variables	11	Explain how quantitative variables were handled in the analyses. If applicable, describe which groupings were chosen and why<br>- Methods; line 90-127
Statistical methods	12	(*a*) Describe all statistical methods, including those used to control for confounding<br>- Methods; line 90-127
		(*b*) Describe any methods used to examine subgroups and interactions<br>- Methods; line 90-127
		(*c*) Explain how missing data were addressed<br>- Not applicable
		(*d*) If applicable, describe analytical methods taking account of sampling strategy<br>- Not applicable
		(*e*) Describe any sensitivity analyses<br>- Not applicable

Results

Participants	13*	(a) Report numbers of individuals at each stage of study—eg numbers potentially eligible, examined for eligibility, confirmed eligible, included in the study, completing follow-up, and analysed<br>- Figure 1
		(b) Give reasons for non-participation at each stage<br>- Figure 1
		(c) Consider use of a flow diagram<br>- Figure 1
Descriptive data	14*	(a) Give characteristics of study participants (eg demographic, clinical, social) and information on exposures and potential confounders<br>- Methods; line 52-57
		(b) Indicate number of participants with missing data for each variable of interest<br>- Not applicable
Outcome data	15*	Report numbers of outcome events or summary measures<br>- Results; line 130-160
Main results	16	(*a*) Give unadjusted estimates and, if applicable, confounder-adjusted estimates and their precision (eg, 95% confidence interval). Make clear which confounders were adjusted for and why they were included<br>- Methods; line 110-112, 121-124
		(*b*) Report category boundaries when continuous variables were categorized<br>- Methods; line 90-127
		(*c*) If relevant, consider translating estimates of relative risk into absolute risk for a meaningful time period<br>- Not applicable
Other analyses	17	Report other analyses done—eg analyses of subgroups and interactions, and sensitivity analyses<br>- Results; line 130-160
**Discussion**		
Key results	18	Summarise key results with reference to study objectives<br>- Discussion line 163-169, 189-190, 218-219
Limitations	19	Discuss limitations of the study, taking into account sources of potential bias or imprecision. Discuss both direction and magnitude of any potential bias<br>- Discussion line 170-188
Interpretation	20	Give a cautious overall interpretation of results considering objectives, limitations, multiplicity of analyses, results from similar studies, and other relevant evidence<br>- Conclusions 232- 239
Generalisability	21	Discuss the generalisability (external validity) of the study results<br>- Not applicable
**Other information**		
Funding	22	Give the source of funding and the role of the funders for the present study and, if applicable, for the original study on which the present article is based<br>- Not applicable

*Give information separately for exposed and unexposed groups.

Note: An Explanation and Elaboration article discusses each checklist item and gives methodological background and published examples of transparent reporting. The STROBE checklist is best used in conjunction with this article (freely

available on the Web sites of PLoS Medicine at http://www.plosmedicine.org/, Annals of Internal Medicine at http://www.annals.org/, and Epidemiology at http://www.epidem.com/). Information on the STROBE Initiative is available at www.strobe-statement.org.
